# 5-HT_2C_ Receptor Stimulation in Obesity Treatment: Orthosteric Agonists vs. Allosteric Modulators

**DOI:** 10.3390/nu15061449

**Published:** 2023-03-17

**Authors:** Edmund Przegaliński, Kacper Witek, Karolina Wydra, Jolanta H. Kotlińska, Małgorzata Filip

**Affiliations:** 1 Department of Drug Addiction Pharmacology, Maj Institute of Pharmacology Polish Academy of Sciences, Smętna Street 12, 31-343 Krakow, Poland; witek@if-pan.krakow.pl (K.W.); wydra@if-pan.krakow.pl (K.W.); mal.fil@if-pan.krakow.pl (M.F.); 2 Department of Pharmacology and Pharmacodynamics, Medical University of Lublin, Chodźki Street 4a, 20-093 Lublin, Poland; jolanta.kotlinska@umlub.pl

**Keywords:** antiobesity drugs, obesity, PAMs, 5-HT_2C_ receptor agonists

## Abstract

Obesity is a substantial health and economic issue, and serotonin (5-hydroxytryptamine, 5-HT) is an important neurotransmitter system involved in the regulation of body weight. The 5-HT_2C_ receptors (5-HT_2C_Rs), one of 16 of the 5-HT receptor (5-HTRs) subtypes, play a significant role in food intake and body weight control. In this review, we focused on the 5-HTR agonists, such as fenfluramines, sibutramine, and lorcaserin, which act directly or indirectly at 5-HT_2C_Rs and have been introduced into the clinic as antiobesity medications. Due to their unwanted effects, they were withdrawn from the market. The 5-HT_2C_R positive allosteric modulators (PAMs) can be potentially safer active drugs than 5-HT_2C_R agonists. However, more in vivo validation of PAMs is required to fully determine if these drugs will be effective in obesity prevention and antiobesity pharmacology treatment. Methodology strategy: This review focuses on the role of 5-HT_2C_R agonism in obesity treatment, such as food intake regulation and weight gain. The literature was reviewed according to the review topic. We searched the PubMed and Scopus databases and Multidisciplinary Digital Publishing Institute open-access scientific journals using the following keyword search strategy depending on the chapter phrases: (1) “5-HT_2C_ receptor” AND “food intake”, and (2) “5-HT_2C_ receptor” AND “obesity” AND “respective agonists”, and (3) “5-HT_2C_ receptor” AND “PAM”. We included preclinical studies (only present the weight loss effects) and double-blind, placebo-controlled, randomized clinical trials published since the 1975s (mostly related to antiobesity treatment), and excluded the pay-walled articles. After the search process, the authors selected, carefully screened, and reviewed appropriate papers. In total, 136 articles were included in this review.

## 1. Introduction

Overweight and obesity are global epidemics affecting more than 2.5 billion people worldwide. Overweight and obesity are related to a body mass index (BMI) >25 kg/m^2^ and >30 kg/m^2^, respectively, and result in substantial health and economic problems. Importantly, obesity is associated with several comorbidities, including chronic diseases, such as diabetes (type 2), cardiovascular pathologies (atherosclerosis, hypertension, and myocardial infarction), stroke, and different kinds of cancers. Thus, obesity is responsible for approximately 15% of human mortality [1]. Therefore, respective medications for obesity are important and are still being investigated in several laboratories.

Serotonin (5-hydroxytryptamine, 5-HT) is a key monoaminergic neurotransmitter in the central nervous system. Mammalian 5-HT is synthesized from the amino acid L-tryptophan by tryptophan hydroxylase to 5-hydroxy-L-tryptophan, and finally by aromatic amino acid decarboxylase enzymes. The 5-HT controls numerous physiological functions, such as thermoregulation, motor activity, wakefulness, cognitive function, and mood [2]. The 5-HT plays a crucial role in food intake modulation and body weight control. In fact, manipulations at a presynaptic level to reduce 5-HT function (e.g., after central administration of 5,7-dihydroxytryptamine, which is a 5-HT neurotoxin) or to enhance 5-HT tone (after 5-hydroxytryptophan, which is the precursor of 5-HT or fenfluramine that releases and inhibits the uptake of 5-HT) provoke an increase or decrease in food intake, respectively [3,4,5].

The multiplicity of 5-HT effects is controlled by a family of 5-HT receptor (5-HTRs) classes, 5-HT_1-7_R [6]. Preclinical studies using 5-HT_2C_R knockout demonstrated significantly higher food intake and body weight gain [7,8], while several selective or non-selective 5-HT_2C_R agonists caused anorectic effects [9]. In addition, the 5-HT_2C_R antagonist can increase weight gain and food intake [10,11]. This suggests that the 5-HT_2C_R may be considered the principal therapeutic target of antiobesity drugs among 5-HT signaling.

The 5-HT_2C_Rs are seven transmembrane spanning helices G protein-coupled receptors (GPCRs) with three extracellular, three intracellular loops, and an extracellular amino-terminus. The 5-HT_2C_Rs primarily are distributed postsynaptically in the central nervous system and are especially present in the epithelial cells of the choroid plexus, limbic areas, hippocampal regions, the amygdala, and basal ganglia [12]. The latter receptors are located on neuronal cell types expressed acetylcholine, dopamine, and gamma-aminobutyric acid neurotransmitters. The 5-HT_2C_Rs activate the phospholipase Cβ via Gα^q/11^ protein and are coupled to phospholipase C in neurons and choroid plexus, and their activation leads to a cellular accumulation of inositol 1,4,5-triphosphate and diglyceride. In addition, 5-HT_2C_Rs have also been shown to regulate ion channels and transport processes as well as activate other downstream effectors [6].

Nowadays, when obesity and overweight are epidemics, the effectiveness of medications is necessary and must be investigated. Whereas the 5-HT system plays a crucial role in food intake and body weight control, 5-HT_2C_Rs are one of the therapeutic targets of antiobesity drugs. Our study aimed to show preclinical research and systematize the obesity treatment efficacy of 5-HT_2C_R agonists in double-blind, placebo-controlled, and randomized clinical trials. Besides, we focused on the future perspective of newly synthesized pharmaceutical molecules, with 5-HT_2C_R activity modulation, as a potential antiobesity treatment.

## 2. 5-HT_2C_ Receptor Action in Food Intake Regulation

The hypothalamus is the most important brain region involved in the central control of feeding and energy expenditure. Within the hypothalamus, the arcuate nucleus controls food intake and metabolism [13]. Functionally, the arcuate nucleus neurons are divided into two antagonistic groups that express specific neuropeptides and elicit disparate physiological actions. The first group consists of orexigenic neurons that express neuropeptide Y and agouti-related peptide to stimulate appetite. The second, anorexigenic group of neurons suppresses appetite with pro-opiomelanocortin (POMC) [14,15]. The modulation of feeding behavior via local 5-HT signaling acts mainly by an anorexigenic effect on POMC neurons. They are controlled by excitatory projections from the raphe nuclei 5-HT-reactive neurons stimulate the POMC-expressing arcuate nucleus neurons [16].

Approximately 25% of adult mouse POMC neurons express the 5-HT_2C_Rs, but activation of POMC neurons via their receptor has been shown to account for the anorexigenic effects of several 5-HT drugs [17]. It has been demonstrated that 5-HT_2C_Rs expressed in hypothalamic POMC/cocaine amphetamine-regulated transcript neurons are required to control energy and glucose homeostasis, implicating POMC neurons as the target for the effect of 5-HT_2C_R agonists on weight-loss induction and improved glycemic control [6,18]. Moreover, 5-HT_2C_R activation in POMC neurons both induces the *Pomc* mRNA expression and increases POMC neuronal activity [19]. Notably, the 5-HT_2C_R knockout phenotype in POMC neurons provokes hyperphagia, sensitizes mice to diet-induced obesity, and directly dysregulates glucose homeostasis [18]. Moreover, selectively reintroducing the 5-HT_2C_R to POMC neurons in whole-body 5-HT_2C_R knockout mice is sufficient to completely negate the knockout phenotype [20]. Finally, 5-HT_2C_Rs re-expression solely in POMC mice neurons is sufficient to mediate the effects of 5-HT drugs on food intake [21].

Food intake is also regulated by the reward brain system (the mesocorticolimbic pathway) structures, such as the ventral tegmental area, nucleus accumbens, prefrontal cortex, hippocampus, and amygdala [22,23]. Changes in reward processing are hypothesized to play a crucial role in the onset and maintenance of binge eating. Binge eating disorder is related to recurrent episodes of frequently consuming unusually large amounts of food and feeling unable to stop eating. Binge eating disorder is also related to its frequent comorbidity, obesity, as well as complications of being overweight [24]. Similar to addictive substances, such as cocaine, food activates the ventral tegmental area–nucleus accumbens pathways and stimulates pleasure by increasing the release of dopamine [25,26]. Moreover, a subset of dopamine neurons of the ventral tegmental area has recently been demonstrated as a key stimulator of binge-like eating behavior in mice [27]. At the same time, 5-HT cell bodies, projecting from the dorsal raphe nuclei to the ventral tegmental area and the nucleus accumbens, and a subset of dopamine and gamma-aminobutyric acid neurons in the ventral tegmental area express the 5-HT_2C_Rs [28,29]. Generally, the activation of 5-HT_2C_Rs plays an inhibitory role in the regulation of reward-related behavior by inhibiting release in certain areas of the brain [30,31]. Activation of ventral tegmental area 5-HT_2C_R-expressing neurons significantly reduces homeostatic feeding in mice [32].

## 3. 5-HT_2C_ Receptor Agonists in Control over Food Intake Preclinical Research

Functionally, the 5-HTR agonists are drugs that orthosterically bind to one (=selective agonist) or more (=nonselective agonist) subtypes of 5-HTR. Direct or indirect (through additional mechanisms) 5-HTR activation by agonists provides a similar response to the intended activation by endogenous 5-HT (Table 1). In rodents, the 5-HT_2C_R agonist drugs reduce food intake in a manner consistent with an enhancement of satiety by action in the 5-HT_2C_R binding site in the brain hypothalamus.

### 3.1. Piperazine Derivatives

The first reports showing that the stimulation of 5-HT_2C_Rs is responsible for the reduction of food intake were based on experiments performed with piperazine derivatives (m-chlorophenyl piperazine; m-CPP and tri-fluoromethylphenylpiperazine; TFMPP), which are nonselective 5-HT receptor agonists (Table 1). These compounds reduced food intake in rodents, and such an effect did not appear in mice lacking 5-HT_2C_Rs [7] or in rats pretreated with 5-HT_2C_R antagonists [37]. It has also been shown that piperazine 5-HT receptor agonists (especially m-CPP) reduce hepatic glucose production and insulin tolerance, and both effects are independent of the reduction in food intake [38]. Other authors presented evidence that the effect of m-CPP and other compounds acting via 5-HT_2C_Rs increases satiety, reduces food intake, does not affect energy expenditure, and decreases body weight [39]. It should also be emphasized that hypophagia, an effect depending on the activation of 5-HT_2C_Rs, is also related to an increase in POMC release in the nucleus arcuatus of the hypothalamus and stimulation of melanocortin receptors in the paraventricular nucleus (PVH) [38,40]. In contrast to the above-mentioned piperazine derivatives, several compounds with different chemical structures that have been previously described are selective 5-HT_2C_R agonists that reduce food intake in rodents. In fact, Org 37684, Ro 60-175, PNU 22394, VER 3323, YM 348, and WAY 163909 have been found to induce hypophagia, which is reduced or blocked by 5-HT_2C_R antagonists [37,41,42,43,44]. Interestingly, the hypophagia induced by 5-HT_2C_R agonists seems to be selective. There is a lack of effect on water intake, and tolerance is not induced after chronic administration. Furthermore, some of these compounds (e.g., PNU 22394) appeared to be clinically active [37].

### 3.2. Fenfluramines

Acting at the presynaptic level, fenfluramine and d-fenfluramine are indirect agonists of 5-HT receptors (Table 1), among which the 5-HT_2C_R are mostly required for the antiobesity effect. Accordingly, it has been demonstrated that, in 5-HT_2C_R knockout mice, the anorexic effect of d-fenfluramine was not induced [21,45]. Furthermore, the hypophagic effect of d-fenfluramine was blocked by 5-HT_2C_R antagonists [39,46]. Preclinical studies show that low (2.5 or 3 mg/kg) and high (10 mg/kg) doses of fenfluramine or d-fenfluramine reduced food intake and decreased body weight in rat models [47,48,49]. Moreover, in the case of cue-induced relapse, d-fenfluramine (3 mg/kg) blocked the reinstatement of lever pressing to food-seeking behavior in male rats [50].

### 3.3. Sibutramine

Similar to the antiobesity effect of fenfluramines, another selective inhibitor of presynaptic reuptake of the 5-HT and noradrenaline (NA) is β-phenethylamine—sibutramine (Table 1). Sibutramine action has also been suggested to be connected to the activation of 5-HT_2C_R and enhancement of POMC [51,52]. Sibutramine (3–10 mg/kg) treatment rats showed reduced food intake and decreased body weight [53,54,55,56,57,58]. Interestingly, intraperitoneal injections of sibutramine (0.5–3.0 mg/kg) reduced feeding on a high fat/high sucrose diet across 2-h feeding sessions, but bilateral injections of sibutramine (2.0–10.0 μg) into either the PVN or the medial nucleus accumbens shell increased the food intake of the sweetened fat diet [59].

### 3.4. Lorcaserin

A series of new 5-HT_2C_R agonists, as derivatives of 3-benzazepine, were synthesized by Smith et al. [60]. The most potent and selective compound was (1R)-8-chloro-2,3,4,5-tetrahydro-1-methyl-1H-3-benzazepine (APD 356, lorcaserin) (Table 1). The authors performed in vitro (effect on turnover of [3H]phosphoinositol) and in vivo (effect on food intake and body weight after acute or chronic *per os* administration of lorcaserin) experiments. The results demonstrated its 5-HT_2C_R selectivity (versus 5-HT_2A_ and 5-HT_2B_ sharing sequence homology) as well as a reduction in food intake after acute administration and reductions in both food intake and body weight after 28 days of treatment. The effect of lorcaserin on food intake has been examined in different animal models of obesity. Some authors used a regular diet [60,61], and other investigators evaluated lorcaserin in rats maintained on a high-fat diet [36,62,63]. Lorcaserin treatment rats (1–2 mg/kg) reduced the percentage of body weight by selective reduction of body fat mass [36,60]. Moreover, Xu et al. performed their experiments using binge-like eating and hunger-driven feeding in mice, where lorcaserin reduced food intake [27]. Lorcaserin also reversed the binge-like feeding observed following stimulation of the nucleus accumbens μ-opioid receptors and blocked nucleus accumbens μ-opioid enhancement of fat intake [64]. The simple antiobesity effect of lorcaserin may be related to the 5-HT_2C_Rs location in brain structures associated with food intake control. It has been shown that the stimulation of such receptors expressed in POMC neurons induces anorexia [20,65], and the stimulation of 5-HT_2C_Rs located on the brain patterning transcription factor single-minded 1 expressing neurons in the PVH increases food intake in mice [66,67].

Other studies showed a reduction in glucose consumption [61] and decreased binge food intake in rats [63] after lorcaserin (1 or 3 mg/kg) treatment. In addition, the above-mentioned antiobesity activity of 5-HT_2C_R agonists is related to increased satiety and decreased food intake. Such effects of lorcaserin can also be connected with its inhibitory effect on motivation and impulsivity [68]. In some experiments, the effect of lorcaserin on glycemic control has been shown. It reduced the production of glucose and increased the sensitivity to insulin. However, it did not affect the secretion of insulin. The above effects were independent of weight loss [69]. Importantly, single-dose pharmacokinetic studies have shown rapid absorption, high oral bioavailability, and a moderate half-life of lorcaserin [60].

## 4. Clinical Effects of 5-HT_2C_ Receptor Agonist Drugs

### 4.1. Fenfluramines

At the beginning of 1970, fenfluramine and d-fenfluramine (an active dextro-rotatory stereoisomer of fenfluramine) were the first antiobesity drugs acting via the 5-HT system to be introduced on the market. In clinical trials, d-fenfluramine showed dose- and time-dependent weight loss (Table 2). In short-term treatment (3–6 months), a higher dose (30–60 mg) of d-fenfluramine led to greater weight loss than low doses (10–15 mg), compared to placebo patients. Long-term treatment (12 months or more) showed greater weight loss in d-fenfluramine patients compared to short-term treatment. Despite the efficacy of fenfluramine or d-fenfluramine in anorexic action, undesirable effects have been observed in obese patients.

In several clinical trials, cardiovascular and heart valve disorders were observed. Echocardiography performed 12 months after the initiation of fenfluramine-phentermine therapy in twenty-four women demonstrated unusual heart valvular morphology and regurgitation in all patients. Moreover, 30% of them also had newly documented pulmonary hypertension [92]. Another study reported that, of 233 obese patients who took appetite suppressants containing d-fenfluramine, 12.7% of them had a prevalence of cardiac valvular insufficiency [93]. Moreover, after the echocardiography of 200 patients with a history of exposure to anorectic medications, significant aortic valve regurgitation was observed in 14% of patients exposed to d-fenfluramine [94]. 

Other authors showed occurrences of multivalvular disease and pulmonary hypertension after fenfluramine treatment [95,96,97,98]. Nine controlled studies of fenfluramine showed mild or greater aortic valve regurgitation in 9.6% of treated almost 3300 patients compared with 3.9% of 2000 control subjects [99]. Besides the cardiovascular problems, the most common events reported during d-fenfluramine (15 mg twice daily) treatment were diarrhea, asthenia, dry mouth, headache, and tiredness. Side effects were reported significantly more frequently in patients receiving dexfenfluramine 30 mg twice daily than in those receiving 15 mg twice daily [100]. 

In 1997, fenfluramines were withdrawn from clinical use due to adverse side effects, such as cardiac valvulopathy and pulmonary hypertension. The most likely explanation for fenfluramine-associated valvulopathy is the activation of 5-HT_2B_Rs by norfenfluramine, being a fenfluramine metabolite. Fenfluramines bind weakly to 5-HT_2A_Rs, 5-HT_2B_Rs, and 5-HT_2C_Rs. Norfenfluramine exhibited a high affinity for 5-HT_2B_Rs, and 5-HT_2C_Rs, and a more moderate affinity for 5-HT_2A_Rs [33]. In cells expressing recombinant 5-HT_2B_Rs, norfenfluramine potently stimulated the hydrolysis of inositol phosphates, increased intracellular Ca^2+^, and activated the mitogen-activated protein kinase cascade, the latter of which has been linked to mitogenic actions of the 5-HT_2B_Rs. This 5-HT_2B_Rs action is important for heart valvulopathy occurrence. Moreover, the level of 5-HT_2B_Rs and 5-HT_2A_Rs transcripts in heart valves was at least 300-fold higher than the levels of the 5-HT_2C_R transcript, which were barely detectable [101]. 

### 4.2. Sibutramine

Sibutramine was approved by the U.S. Food and Drug Administration (FDA) in 1997 for weight loss in the short- and long-term treatment of obesity. Sibutramine has dose- and time- dependent antiobesity effectiveness, as evidenced in clinical studies (Table 2). Compared to placebo-treated patients, higher doses (15–30 mg) of sibutramine result in greater weight loss than low doses (10 mg). Moreover, sibutramine efficacy in reducing body weight in a long-term period (over one year) was greater than in short-term treatment (six months). However, the Sibutramine Cardiovascular Outcomes (SCOUT) trial showed that, at 12 months but not six months of sibutramine treatment, patients with marked lower baseline blood pressure had the tendency to increase [102]. In some other clinical studies, sibutramine did not increase blood pressure in obese patients [103,104]. Besides, patients taking sibutramine experienced a significant increase in heart rate [83,105,106,107,108,109], but without difference in the overall status of the cardiac valves compared to placebo subjects [105,110,111,112]. Otherwise, sibutramine’s most frequently encountered adverse effects were constipation, dry mouth, headache, insomnia, and nausea.

In 2010, the FDA decided to withdraw sibutramine from the market as a consequence of increased primary outcome events (POEs), such as nonfatal myocardial infarction, nonfatal stroke, resuscitation after cardiac arrest, or cardiovascular death. Indeed, the risk of POEs was increased by 16% in the sibutramine treatment group as compared with the placebo [80]. Nevertheless, other results from SCOUTs did not show an overall deleterious effect of treatment but an increase in POEs in subjects with a history of cardiovascular disease and type 2 diabetes [113,114]. Unexpectedly, sibutramine therapy in obese or overweight high-risk patients induced significant mean reductions for all blood lipids [115]. Although sibutramine and its metabolites exhibit low affinity for 5-HT_1A-B_Rs and 5-HT_2A_Rs, these receptors are reported as a possible target for the causation of unwanted cardiovascular effects [116]. 

### 4.3. Lorcaserin

In 2012, the FDA approved lorcaserin on the market as a weight loss medication. The results of several clinical studies have shown its antiobesity effect (Table 2). Despite the time of treatment, lorcaserin (10 mg once and twice daily) caused greater weight loss than with a placebo. The most commonly reported adverse effects were headache, nausea, dizziness, and fatigue, but generally, lorcaserin was well-tolerated. In contrast, the unwanted effects of fenfluramines and sibutramine, in randomized, double-blind, placebo-controlled, multinational clinical trials of lorcaserin treatment, were not associated with any increase in cardiovascular events among patients with high cardiovascular risk [88]. Interestingly, lorcaserin treatment for 6–13 months showed an improvement in metabolic and cardiovascular parameters in obese patients [91,117,118]. It has also been reported that this drug decreases the risk of developing diabetes [119]. At the same time, compared to a placebo, lorcaserin did not affect blood pressure, heart rate, or heart valves [120,121,122]. 

However, the US Food and Drug Administration Adverse Event Reporting System (FAERS) database scrutinization identified that lorcaserin medication was associated with a significantly greater valvular disorder [123]. Only in overweight and obese patients with renal dysfunction, lorcaserin treatment barely increased cardiovascular disease risk [124]. At the same time, the results of several clinical studies have shown its modest antiobesity effects, and lorcaserin’s position on the market slowly declined. Moreover, patients treated with lorcaserin were diagnosed with more frequent occurrences of cancer. Indeed, incidences of malignancies during lorcaserin pharmacotherapy (though not different from the respective placebo groups) were the reason that it was withdrawn as an antiobesity medication in 2020 [125,126,127].

## 5. Positive-Allosteric Modulators of the 5-HT_2C_R

**Figure 1:**
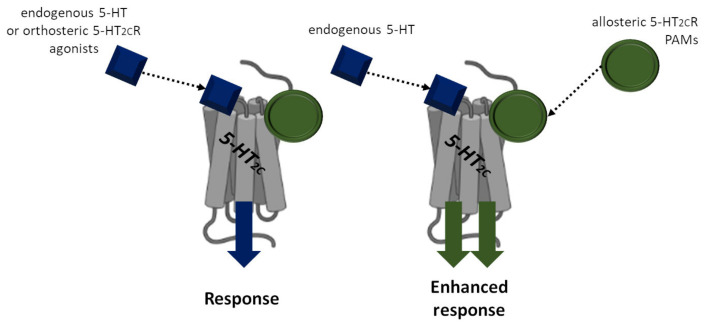
Mechanisms of the 5-HT_2C_R function modulation by agonists and positive-allosteric modulators (PAMs). The 5-HT_2C_R agonists bind to the same orthosteric sites as endogenous serotonin (5-HT) at the 5-HT_2C_R to evoke a response. Opposed to the 5-HT_2C_R agonists, the 5-HT_2C_R PAMs bind to allosteric sites at the 5-HT_2C_R, enhancing the affinity and efficacy of the 5-HT_2C_R response by 5-HT.

The 5-HT_2C_R is not only an antiobesity drug target, but also a binding site for positive-allosteric modulators (PAMs). PAMs are groups of substances that increase the affinity and/or efficacy of respective receptor ligands (Figure 1). A potential PAM therapeutic role is the ability to potentiate the effect of an endogenous cognate ligand or other probes interacting orthosterically by binding at a distinct, allosteric, receptor recognition site [128]. Thereafter, several new molecules related to 5-HT_2C_R PAMs were pharmacologically evaluated. Recently, there have been a few papers showing 5-HT_2C_R PAM to be potentially safer antiobesity drugs. Garcia-Carceles et al. [129] screened a chemical library from Vivia Biotech using the ExviTech platform and validated and synthesized analogues of Compound 5 (VA240). Among 35 synthesized akins, the most interesting was Compound 11 (PAM 11: (N-[(1-benzyl-1H-indol-3-yl)methyl]pyridin-3-amine, VA012). This compound dose-dependently enhanced 5-HT efficacy (highest potentiation—by 35% at 10 µM—of 5-HT-induced inositol monophosphate release in 5-HT_2C_R HeLa cells), displayed low binding competition with 5-HT or other orthosteric ligands, and did not exhibit significant off-target activities. Importantly, PAM 11 and WAY 161503 (an agonist of 5-HT_2C_Rs used as a reference drug) were administered acutely to reduce food intake in rats, and PAM 11 acted with higher efficacy and with more prolonged action. Moreover, PAM 11-evoked food intake inhibition was not eliminated after pretreatment with the 5-HT_2A_R antagonist ketanserin. These findings indicate that the effect of PAM 11 was not related to the activation of 5-HT_2A_Rs and suggest that it has no direct action at the orthosteric site of the 5-HT_2C_R. Furthermore, PAM 11 administered subchronically (seven days) in a restricted food access model also reduced both food intake and body weight gain in rats. Finally, after combined treatment with PAM 11 and sertraline (a 5-HT reuptake inhibitor) administered in a dose-induced mild feeding suppression, potentiation of feeding reduction was demonstrated. This is clear evidence for the allosteric potentiation of 5-HT-induced anorexia, especially 5-HT_2C_R-induced anorexia [129]. Another study synthesized a series of N-heterocycle imidazole-linked phenyl cyclopropyl methanone molecules. One of three active 5-HT_2C_R allosteric modulator molecules, Compound 58, showed unique and beneficial dual characteristics. Compound 58 selectively exhibited PAM response at 5-HT_2C_Rs and negative-allosteric modulator at 5-HT_2B_Rs, which is rarely reported for allosteric modulators. From the point of view of 5-HT_2C_R agonist’s unwanted cardiovascular effects (depending on 5-HT_2B_Rs activation), the negative 5-HT_2B_Rs modulation is a unique property. Besides, at the same dose, both Compound 58 and lorcaserin reduced food intake in rats [130]. 

## 6. Conclusions and Perspectives 

This paper reviews the preclinical and clinical efficacy of 5-HT_2C_R agonists in obesity treatment. Moreover, we showed evidence of new therapeutic molecules, based on 5-HT_2C_R targets, as potential antiobesity drugs.

In preclinical studies, nonselective or more selective 5-HT_2C_R agonists showed effectiveness in decreasing body weight, reducing food intake, and inhibiting food seeking-behavior. Consequently, 5-HT_2C_R drugs, such as fenfluramines, sibutramine, and lorcaserin, were approved in the trade as obesity pharmacology medications. Clinical trials confirmed 5-HT_2C_R agonist’s action in reduced food intake and decreased body weight in patients with BMI >25 kg/m^2^ or an ideal body weight of 120–180%. However, after a few years, the 5-HT_2C_R agonist’s position on the market slowly declined, until being withdrawn from the market due to unwanted effects (higher risk of cardiac valvular abnormalities) occurrence. Nonetheless, the above-mentioned drugs have contributed to understanding the mechanisms of 5-HTR action that permitted the design of new selective molecules. Herein, we showed that 5-HT_2C_R PAM intensifies 5-HT action for reduced food intake and decreased weight loss in a few in vivo studies. Moreover, we presented selective 5-HT_2C_R PAM without 5-HT_2B_R-linked cardiac valve affinity that offers a new angle into the pharmacological potential safer in obesity treatment.

Nowadays, more attention is being paid to precision medicine that offers new grounds for obesity prevention and targeting correct treatment based on genetic, pharmacogenomic, and environmental factors [131,132,133]. Recent human studies underline the connectivity between 5-HT availability and BMI as a predictor of obesity treatment success [134]. Furthermore, the loss of 5-HT_2C_R function due to mutation can predispose humans to obesity. Thus, sequencing genes encoding the 5-HT_2C_R should be included in diagnostic panels for obesity [135]. Currently, a series of highly specific 5-HT_2C_R PAM selectively increase in vitro 5-HT efficacy [136]. This elevates the possibility of 5-HT_2C_R PAM being used in a more directed way as precision medicine.

In conclusion, we underline the role of 5-HT_2C_R as a crucial therapeutic target in obesity treatment. Further, 5-HT_2C_R PAM discovery and syntheses are recommended. Furthermore, 5-HT_2C_R PAM optimization and clinical weight loss validation are necessary for successful antiobesity treatment and to reveal the full therapeutic potential of PAM.

## Figures and Tables

**Table 1:** Description of antiobesity drugs affinity to 5-HT_1-2_ receptor (5-HT_1-2_R) subtypes, type of binding and mechanisms of 5-HT_2C_ receptor (5-HT_2C_R) activation.

Drug	Affinity to 5-HT_1-2_R Subtypes *	Selectivity to 5-HT_2C_R	Mechanism of 5-HT_2C_R Activation
piperazine derivatives (e.g., m-chlorophenyl piperazine)	5-HT_2B_ ≥ 5-HT_2C_ > 5-HT_2A_ > 5-HT_1A_ ≥ 5-HT_1B_ [33]	nonselective ligand	indirect agonism (affinity to 5-HT transporter and followed by 5-HT reuptake inhibition or 5-HT release enhancement)
fenfluramines	5-HT_2B_ ≥ 5-HT_2C_ > 5-HT_2A_ [34]	nonselective ligand	indirect agonism (affinity to 5-HT transporter and followed by 5-HT reuptake inhibition)
sibutramine	5-HT_1A_ = 5-HT_1B_ = 5-HT_2A_ = 5-HT_2C_ [35]	nonselective ligand	indirect agonism (affinity to 5-HT transporter and followed by 5-HT reuptake inhibition)
lorcaserin	5-HT_2C_ > 5-HT_2A_ > 5-HT_2B_ [36]	selective ligand	direct agonism

* mostly important for food intake and unwanted effects.

**Table 2:** Efficacy of 5-HT_2C_ receptor agonist properties in double-blind, placebo-controlled, randomized clinical obesity trials. The patient’s inclusion criteria are increased body mass index (BMI; >25 kg/m^2^) or ideal body weight (IBW; 120–180%). The mean weight loss (kg or %) of the drug group (D) is compared to the placebo group (P). Multicenter studies were marked by an asterisk (*). QD—once-a-day treatment; BID—two times-a-day treatment.

Drug	Dosage (mg)	Duration (Months)	Patients Completed Study D|P	Key Inclusion Criteria (BMI > 25 kg/m^2^ or IBW 120–180%)	Mean Weight Loss D vs. P (kg or %)	Ref.
d-fenfluramine	10 BID	3	85|85 *	120–180%	2.79 vs. 2.83 kg	[70]
	15 BID		168|169	120–180%	5.84 vs. 1.85 kg	[71]
			12|12	28–35 kg/m^2^	3.1 ± 2.3 vs. 0.1 ± 1.2 kg	[72]
		12	404|418 *	≥120%	9.82 vs. 7.15 kg	[73]
	30 QD or BID	3	82|85 *	120–180%	5.63 vs. 2.83 kg	[70]
			30|30	120–180%	4.6 ± 1.6 kg vs. no changed	[74]
		12	36|39	≥135%	12.8 vs. 8.6 kg	[75]
	60 BID	3	87|85 *	120–180%	7.23 vs. 2.83 kg	[70]
Sibutramine	5 QD	2	19|20	130–180%	2.9 ± 2.3 vs. 1.4 ± 2.1 kg	[76]
		3	56|59 *	27–40 kg/m^2^	2.4 ± 0.5 vs. 1.4 ± 0.5 kg	[77]
		6	107|87 *	30–40 kg/m^2^	3.7 vs. 1.3 kg	[78]
	10 QD	3	59|59 *	27–40 kg/m^2^	5.1 ± 0.5 vs. 1.4 ± 0.5 kg	[77]
		6	99|87 *	30–40 kg/m^2^	5.7 vs. 1.3 kg	[78]
			104|94 *	≥30 and ≤40 kg/m^2^	8.2 vs. 3.9 kg	[79]
		40	2933|2825 *	25–45 kg/m^2^	1.7 vs. +0.7 kg	[80]
	15 QD	3	62|59 *	27–40 kg/m^2^	2 4.9 ± 0.5 vs. 1.4 ± 0.5 kg	[77]
		6	98|87 *	30–40 kg/m^2^	7.0 vs. 1.3 kg	[78]
		12	281|80	±44 kg/m^2^	6.5 ± 0.31 vs. 1.9 ± 0.56 kg	[81]
			68|64 *	>27 kg/m^2^	5.5 ± 0.6 vs. 0.2 ± 0.5 kg	[82]
		~14	114|103 *	≥30 and <40 kg/m^2^	8.1 ± 8.2 vs. 5.1 ± 6.5 kg	[83]
	20 QD	2	21|20	130–180%	5.0 ± 2.7 vs. 1.4 ± 2.1	[76]
		6	96|87 *	30–40 kg/m^2^	8.2 vs. 1.3 kg	[78]
			151|152 *	≥27 kg/m^2^	4.9 vs. 0.6 kg	[84]
		12	62|64 *	>27 kg/m^2^	8.0 ± 0.9 vs. 0.2 ± 0.5 kg	[82]
	30 QD	6	101|87 *	30–40 kg/m^2^	9.0 vs. 1.3 kg	[78]
Lorcaserin	10 QD or 10 BID	~2	29|28	27–45 kg/m^2^	3.8 ± 0.4 vs. 2.2 ± 0.5 kg	[85]
		3	86 or 77|88 *	30–45 kg/m^2^	1.8 or 3.6 vs. 0.3 kg	[86]
		6	59|53 *	≥33 and ≤55 kg/m^2^	2.4 ± 0.8 vs. +0.6 ± 0.8 kg	[87]
		12	748|243 *	≥27 kg/m^2^	4.2 vs. 1.4 kg	[88]
		12	75 or 169|157 *	27–45 kg/m^2^	44.7 or 37.5 vs. 16.1%	[89]
			275|684 *	27–45 kg/m^2^	5.81 ± 0.16 vs. 2.16 ± 0.14%	[90]
		13	1800|1550	30–45 kg/m^2^	47.1 vs. 22.6%	[91]
		24	564|684 *	27–45 kg/m^2^	7.0 ± 0.2 vs. 3.0 ± 0.2%	[90]
		40	748|243 *	≥27 kg/m^2^	4.0 kg vs. 2.1 kg	[88]
	15 QD	3	82|88 *	30–45 kg/m^2^	2.6 vs. 0.3 kg	[86]

## Data Availability

Not applicable.

## References

[1] Hurt R.T., Mundi M.S., Ebbert J.O. (2018). Challenging Obesity, Diabetes, and Addiction: The Potential of Lorcaserin Extended Release. Diabetes Metab. Syndr. Obes..

[2] Dutton A.C., Barnes N.M. (2006). Anti-Obesity Pharmacotherapy: Future Perspectives Utilising 5-HT_2C_ Receptor Agonists. Drug Discov. Today Ther. Strat..

[3] Saller C.F., Stricker E.M. (1976). Hyperphagia and Increased Growth in Rats After Intraventricular Injection of 5,7-Dihydroxytryptamine. Science.

[4] Blundell J.E., Leshem M.B. (1975). The Effect of 5-Hydroxytryptophan on Food Intake and on the Anorexic Action of Amphetamine and Fenfluramine. J Pharm. Pharmacol..

[5] Duhault J., Malen C., Boulanger M., Voisin C., Beregi L., Schmitt H. (1975). Fenfluramine and 5 Hydroxytryptamine. I: Is Fenfluramine or Norfenfluramine Involved in the Decrease of Brain 5 Hydroxytryptamine?. Arzneim.-Forsch./Drug Res..

[6] Masson J., Emerit M.B., Hamon M., Darmon M. (2012). Serotonergic Signaling: Multiple Effectors and Pleiotropic Effects. Wiley Interdiscip. Rev. Membr. Transp. Signal..

[7] Tecott L.H., Sun L.M., Akana S.F., Strack A.M., Lowenstein D.H., Dallman M.F., Julius D. (1995). Eating Disorder and Epilepsy in Mice Lacking 5-HT^2C^ Serotonin Receptors. Nature.

[8] Nonogaki K., Nozue K., Oka Y. (2006). Hyperphagia Alters Expression of Hypothalamic 5-HT_2C_ and 5-HT_1B_ Receptor Genes and Plasma Des-Acyl Ghrelin Levels in Ay Mice. Endocrinology.

[9] Bello N.T., Liang N.C. (2011). The Use of Serotonergic Drugs to Treat Obesity—Is There Any Hope?. Drug Des. Dev. Ther..

[10] Schuhler S., Clark A., Joseph W., Patel A., Lehnen K., Stratford E., Horan T.L., Fone K.C.F., Ebling F.J.P., Schuhler A. (2005). Involvement of 5-HT Receptors in the Regulation of Food Intake in Siberian Hamsters. J. Neuroendocrinol..

[11] Bonhaus D.W., Weinhardt K.K., Taylor M., Desouza A., Mcneeley P.M., Szczepanski K., Fontana D.J., Trinh J., Rocha C.L., Dawson M.W. (1997). RS-102221: A Novel High Affinity and Selective, 5-HT_2C_ Receptor Antagonist. Neuropharmacology.

[12] Wold E.A., Wild C.T., Cunningham K.A., Zhou J. (2019). Targeting the 5-HT_2C_ Receptor in Biological Context and the Current State of 5-HT_2C_ Receptor Ligand Development. Curr. Top. Med. Chem..

[13] Myers M.G., Olson D.P. (2012). Central Nervous System Control of Metabolism. Nature.

[14] Gropp E., Shanabrough M., Borok E., Xu A.W., Janoschek R., Buch T., Plum L., Balthasar N., Hampel B., Waisman A. (2005). Agouti-Related Peptide–Expressing Neurons Are Mandatory for Feeding. Nat. Neurosci..

[15] Balthasar N., Dalgaard L.T., Lee C.E., Yu J., Funahashi H., Williams T., Ferreira M., Tang V., McGovern R.A., Kenny C.D. (2005). Divergence of Melanocortin Pathways in the Control of Food Intake and Energy Expenditure. Cell.

[16] Van Galen K.A., Ter Horst K.W., Serlie M.J. (2021). Serotonin, Food Intake, and Obesity. Obes. Rev..

[17] Doslikova B., Garfield A.S., Shaw J., Evans M.L., Burdakov D., Billups B., Heisler L.K. (2013). 5-HT_2C_ Receptor agonist Anorectic Efficacy Potentiated by 5-HT_1B_ Receptor agonist Coapplication: An Effect Mediated via Increased Proportion of Pro-Opiomelanocortin Neurons Activated. J. Neurosci..

[18] Berglund E.D., Liu C., Sohn J.W., Liu T., Kim M.H., Lee C.E., Vianna C.R., Williams K.W., Xu Y., Elmquist J.K. (2013). Serotonin 2C Receptors in Pro-Opiomelanocortin Neurons Regulate Energy and Glucose Homeostasis. J. Clin. Investig..

[19] Wyler S.C., Lord C.C., Lee S., Elmquist J.K., Liu C. (2017). Serotonergic Control of Metabolic Homeostasis. Front. Cell. Neurosci..

[20] Roepke T.A., Smith A.W., Rønnekleiv O.K., Kelly M.J. (2012). Serotonin 5-HT_2C_ Receptor-Mediated Inhibition of the M-Current in Hypothalamic POMC Neurons. Am. J. Physiol. Endocrinol. Metab..

[21] Xu Y., Jones J.E., Kohno D., Williams K.W., Lee C.E., Choi M.J., Anderson J.G., Heisler L.K., Zigman J.M., Lowell B.B. (2008). 5-HT_2C_Rs Expressed by Pro-Opiomelanocortin Neurons Regulate Energy Homeostasis. Neuron.

[22] Alonso-Alonso M., Woods S.C., Pelchat M., Grigson P.S., Stice E., Farooqi S., Khoo C.S., Mattes R.D., Beauchamp G.K. (2015). Food Reward System: Current Perspectives and Future Research Needs. Nutr. Rev..

[23] Leenaerts N., Jongen D., Ceccarini J., van Oudenhove L., Vrieze E. (2022). The Neurobiological Reward System and Binge Eating: A Critical Systematic Review of Neuroimaging Studies. Int. J. Eat. Disord..

[24] Amianto F., Ottone L., Abbate Daga G., Fassino S. (2015). Binge-Eating Disorder Diagnosis and Treatment: A Recap in Front of DSM-5. BMC Psychiatry.

[25] Cunningham K.A., Fox R.G., Anastasio N.C., Bubar M.J., Stutz S.J., Moeller F.G., Gilbertson S.R., Rosenzweig-Lipson S. (2011). Selective Serotonin 5-HT(2C) Receptor Activation Suppresses the Reinforcing Efficacy of Cocaine and Sucrose but Differentially Affects the Incentive-Salience Value of Cocaine- vs. Sucrose-Associated Cues. Neuropharmacology.

[26] Price A.E., Anastasio N.C., Stutz S.J., Hommel J.D., Cunningham K.A. (2018). Serotonin 5-HT_2C_ Receptor Activation Suppresses Binge Intake and the Reinforcing and Motivational Properties of High-Fat Food. Front. Pharmacol..

[27] Xu P., He Y., Cao X., Valencia-Torres L., Yan X., Saito K., Wang C., Yang Y., Hinton A., Zhu L. (2017). Activation of Serotonin 2C Receptors in Dopamine Neurons Inhibits Binge-like Eating in Mice. Biol. Psychiatry.

[28] Bubar M.J., Cunningham K.A. (2007). Distribution of Serotonin 5-HT_2C_ Receptors in the Ventral Tegmental Area. Neuroscience.

[29] Bubar M.J., Stutz S.J., Cunningham K.A. (2011). 5-HT_2C_ Receptors Localize to Dopamine and GABA Neurons in the Rat Mesoaccumbens Pathway. PLoS ONE.

[30] De Deurwaerdère P., Navailles S., Berg K.A., Clarke W.P., Spampinato U. (2004). Constitutive Activity of the Serotonin2C Receptor Inhibits In Vivo Dopamine Release in the Rat Striatum and Nucleus Accumbens. J. Neurosci..

[31] Hayes D.J., Mosher T.M., Greenshaw A.J. (2009). Differential Effects of 5-HT_2C_ Receptor Activation by WAY 161503 on Nicotine-Induced Place Conditioning and Locomotor Activity in Rats. Behav. Brain Res..

[32] Valencia-Torres L., Olarte-Sánchez C.M., Lyons D.J., Georgescu T., Greenwald-Yarnell M., Myers M.G., Bradshaw C.M., Heisler L.K. (2017). Activation of Ventral Tegmental Area 5-HT_2C_ Receptors Reduces Incentive Motivation. Neuropsychopharmacology.

[33] Rothman R.B., Baumann M.H., Savage J.E., Rauser L., McBride A., Hufeisen S.J., Roth B.L. (2000). Evidence for Possible Involvement of 5-HT_2B_ Receptors in the Cardiac Valvulopathy Associated With Fenfluramine and Other Serotonergic Medications. Circulation.

[34] Setola V., Dukat M., Glennon R.A., Roth B.L. (2005). Molecular Determinants for the Interaction of the Valvulopathic Anorexigen Norfenfluramine with the 5-HT_2B_ Receptor. Mol. Pharmacol..

[35] Frassetto S.S., Delia Santa Rubio Â., Lopes J.J., Pereira P., Brum C., Khazzaka M., Vinagre A.S. (2006). Locomotor and Peripheral Effects of Sibutramine Modulated by 5-HT_2_ Receptors. Can. J. Physiol. Pharmacol..

[36] Thomsen W.J., Grottick A.J., Menzaghi F., Reyes-Saldana H., Espitia S., Yuskin D., Whelan K., Martin M., Morgan M., Chen W. (2008). Lorcaserin, a Novel Selective Human 5-Hydroxytryptamine2C Agonist: In Vitro and in Vivo Pharmacological Characterization. J. Pharmacol. Exp. Ther..

[37] Bickerdike M. (2003). 5-HT_2C_ Receptor agonists as Potential Drugs for the Treatment of Obesity. Curr. Top. Med. Chem..

[38] Georgescu T., Lyons D., Heisler L.K. (2021). Role of Serotonin in Body Weight, Insulin Secretion and Glycaemic Control. J. Neuroendocrinol..

[39] Hayashi A., Suzuki M., Sasamata M., Miyata K. (2005). Agonist diversity in 5-HT_2C_ receptor-mediated weight control in rats. Psychopharmacology.

[40] Xu Y., Jones J.E., Lauzon D.A., Anderson J.G., Balthasar N., Heisler L.K., Zinn A.R., Lowell B.B., Elmquist J.K. (2010). A Serotonin and Melanocortin Circuit Mediates D-Fenfluramine Anorexia. J. Neurosci..

[41] Martin J.R., Bös M., Jenck F., Moreau J.L., Mutel V., Sleight A.J., Wichmann J., Andrews J.S., Berendsen H.H.G., Broekkamp C.L.E. (1998). 5-HT_2C_ Receptor Agonists: Pharmacological Characteristics and Therapeutic Potential. J. Pharmacol. Exp. Ther..

[42] Kimura Y., Hatanaka K.I., Naitou Y., Maeno K., Shimada I., Koakutsu A., Wanibuchi F., Yamaguchi T. (2004). Pharmacological Profile of YM348, a Novel, Potent and Orally Active 5-HT_2C_ Receptor Agonist. Eur. J. Pharmacol..

[43] Vickers S.P., Benwell K.R., Porter R.H., Bickerdike M.J., Kennett G.A., Dourish C.T. (2000). Comparative Effects of Continuous Infusion of MCPP, Ro 60-0175 and d-Fenfluramine on Food Intake, Water Intake, Body Weight and Locomotor Activity in Rats. Br. J. Pharmacol..

[44] Dunlop J., Sabb A.L., Mazandarani H., Zhang J., Kalgaonker S., Shukhina E., Sukoff S., Vogel R.L., Stack G., Schechter L. (2005). WAY-163909 [(7bR, 10aR)-1,2,3,4,8,9,10,10a-Octahydro-7bH-Cyclopenta-[b][1,4]Diazepino[6,7,1hi]Indole], a Novel 5-Hydroxytryptamine 2C Receptor-Selective Agonist with Anorectic Activity. J. Pharmacol. Exp. Ther..

[45] Vickers S.P., Clifton P.G., Dourish C.T., Tecott L.H. (1999). Reduced Satiating Effect of D-Fenfluramine in Serotonin 5-HT(2C) Receptor Mutant Mice. Psychopharmacology.

[46] Vickers S.P., Dourish C.T., Kennett G.A. (2001). Evidence That Hypophagia Induced by D-Fenfluramine and d-Norfenfluramine in the Rat Is Mediated by 5-HT_2C_ Receptors. Neuropharmacology.

[47] Fisler J.S., Underberger S.J., York D.A., Bray G.A. (1993). D-Fenfluramine in a Rat Model of Dietary Fat-Induced Obesity. Pharmacol. Biochem. Behav..

[48] Clifton P.G., Lee M.D., Dourish C.T. (2000). Similarities in the Action of Ro 60-0175, a 5-HT_2C_ Receptor Agonist, and d-Fenfluramine on Feeding Patterns in the Rat. Psychopharmacology.

[49] Brindley D.N., Hales P., Al-Sieni A.I.I., Russell J.C. (1992). Sustained Decreases in Weight and Serum Insulin, Glucose, Triacylglycerol and Cholesterol in JCR:LA-Corpulent Rats Treated with D-Fenfluramine. Br. J. Pharmacol..

[50] Pratt W.E., Ford R.T. (2013). Systemic Treatment with D-Fenfluramine, but Not Sibutramine, Blocks Cue-Induced Reinstatement of Food-Seeking Behavior in the Rat. Neurosci. Lett..

[51] Burke L.K., Doslikova B., D’Agostino G., Garfield A.S., Farooq G., Burdakov D., Low M.J., Rubinstein M., Evans M.L., Billups B. (2014). 5-HT Obesity Medication Efficacy via POMC Activation Is Maintained During Aging. Endocrinology.

[52] Higgs S., Cooper A.J., Barnes N.M. (2011). Reversal of Sibutramine-Induced Anorexia with a Selective 5-HT(2C) Receptor Antagonist. Psychopharmacology.

[53] Levin B.E., Dunn-Meynell A.A. (2000). Sibutramine Alters the Central Mechanisms Regulating the Defended Body Weight in Diet-Induced Obese Rats. Am. J. Physiol. Regul. Integr. Comp. Physiol..

[54] Hansen H.H., Hansen G., Tang-Christensen M., Larsen P.J., Axel A.M.D., Raben A., Mikkelsen J.D. (2010). The Novel Triple Monoamine Reuptake Inhibitor Tesofensine Induces Sustained Weight Loss and Improves Glycemic Control in the Diet-Induced Obese Rat: Comparison to Sibutramine and Rimonabant. Eur. J. Pharmacol..

[55] Madsen A.N., Hansen G., Paulsen S.J., Lykkegaard K., Tang-Christensen M., Hansen H.S., Levin B.E., Larsen P.J., Knudsen L.B., Fosgerau K. (2010). Long-Term Characterization of the Diet-Induced Obese and Diet-Resistant Rat Model: A Polygenetic Rat Model Mimicking the Human Obesity Syndrome. J. Endocrinol..

[56] Hansen G., Jelsing J., Vrang N. (2012). Effects of Liraglutide and Sibutramine on Food Intake, Palatability, Body Weight and Glucose Tolerance in the Gubra DIO-Rats. Acta Pharmacol. Sin..

[57] Casado A., Rodríguez V.M., Portillo M.P., Macarulla M.T., Abecia L.C., Echevarría E., Casis L. (2003). Sibutramine Decreases Body Weight Gain and Increases Energy Expenditure in Obese Zucker Rats without Changes in NPY and Orexins. Nutr. Neurosci..

[58] Jackson H.C., Needham A.M., Hutchins L.J., Mazurkiewicz S.E., Heal D.J. (1997). Comparison of the Effects of Sibutramine and Other Monoamine Reuptake Inhibitors on Food Intake in the Rat. Br. J. Pharmacol..

[59] Pratt W.E., Connolly M.E. (2010). Contrasting Effects of Systemic and Central Sibutramine Administration on the Intake of a Palatable Diet in the Rat. Neurosci. Lett..

[60] Smith B.M., Smith J.M., Tsai J.H., Schultz J.A., Gilson C.A., Estrada S.A., Chen R.R., Park D.M., Prieto E.B., Gallardo C.S. (2008). Discovery and Structure-Activity Relationship of (1R)-8-Chloro-2,3,4,5-Tetrahydro-1-Methyl-1H-3-Benzazepine (Lorcaserin), a Selective Serotonin 5-HT_2C_ Receptor Agonist for the Treatment of Obesity. J. Med. Chem..

[61] Higgs S., Cooper A.J., Barnes N.M. (2016). The 5-HT_2C_ Receptor Agonist, Lorcaserin, and the 5-HT6 Receptor Antagonist, SB-742457, Promote Satiety; A Microstructural Analysis of Feeding Behaviour. Psychopharmacology.

[62] Higgins G.A., Desnoyer J., van Niekerk A., Silenieks L.B., Lau W., Thevarkunnel S., Izhakova J., Delannoy I.A.M., Fletcher P.J., Delay J. (2015). Characterization of the 5-HT_2C_ Receptor Agonist Lorcaserin on Efficacy and Safety Measures in a Rat Model of Diet-Induced Obesity. Pharmacol. Res. Perspect..

[63] Price A.E., Brehm V.D., Hommel J.D., Anastasio N.C., Cunningham K.A. (2018). Pimavanserin and Lorcaserin Attenuate Measures of Binge Eating in Male Sprague-Dawley Rats. Front. Pharmacol..

[64] Blumenthal S.A., Pratt W.E. (2018). D-Fenfluramine and Lorcaserin Inhibit the Binge-like Feeding Induced by μ-Opioid Receptor Stimulation of the Nucleus Accumbens in the Rat. Neurosci. Lett..

[65] D’Agostino G., Lyons D., Cristiano C., Lettieri M., Olarte-Sanchez C., Burke L.K., Greenwald-Yarnell M., Cansell C., Doslikova B., Georgescu T. (2018). Nucleus of the Solitary Tract Serotonin 5-HT_2C_ Receptors Modulate Food Intake. Cell Metab..

[66] Zhan C., Zhou J., Feng Q., Zhang J.E., Lin S., Bao J., Wu P., Luo M. (2013). Acute and Long-Term Suppression of Feeding Behavior by POMC Neurons in the Brainstem and Hypothalamus, Respectively. J. Neurosci..

[67] Kwon E., Jo Y.H. (2020). Activation of the ARCPOMC→MeA Projection Reduces Food Intake. Front. Neural Circuits.

[68] Higgins G.A., Zeeb F.D., Fletcher P.J. (2017). Role of Impulsivity and Reward in the Anti-Obesity Actions of 5-HT 2C Receptor Agonists. J. Psychopharmacol..

[69] Burke L.K., Ogunnowo-Bada E., Georgescu T., Cristiano C., de Morentin P.B.M., Valencia Torres L., D’Agostino G., Riches C., Heeley N., Ruan Y. (2017). Lorcaserin Improves Glycemic Control via a Melanocortin Neurocircuit. Mol. Metab..

[70] Marbury T.C., Angelo J.E., Michael Gulley R., Krosnick A., Sugimoto D.H., Zellner S.R. (1996). A Placebo-Controlled, Dose-Response Study of Dexfenfluramine in the Treatment of Obese Patients. Curr. Ther. Res..

[71] Lucas C.P., Sandage B.W. (1995). Treatment of Obese Patients with Dexfenfluramine: A Multicenter, Placebo-Controlled Study. Am. J. Ther..

[72] Drent M.L., Adèr H.J., van der Veen E.A. (1995). The Influence of Chronic Administration of the Serotonin Agonist Dexfenfluramine on Responsiveness to Corticotropin Releasing Hormone and Growth Hormone-Releasing Hormone in Moderately Obese People. J. Endocrinol. Investig..

[73] Guy-Grand B., Crepaldi G., Vre P.L., Apfelbaum M., Gries A., Turner P. (1989). International Trial of Long-Term Dexfenfluramine in Obesity. Lancet.

[74] Lafreniere F., Lambert L.J., Rasio E., Serri O. (1993). Effects of Dexfenfluramine Treatment on Body Weight and Postprandial Thermogenesis in Obese Subjects. A Double-Blind Placebo-Controlled Study. Int. J. Obes. Relat. Metab. Disord..

[75] Mathus-Vliegen E.M.H., van de Voorde K., Kok A.M.E., Res A.M.A. (1992). Dexfenfluramine in the Treatment of Severe Obesity: A Placebo-Controlled Investigation of the Effects on Weight Loss, Cardiovascular Risk Factors, Food Intake and Eating Behaviour. J. Intern. Med..

[76] Weintraub M., Rubio A., Golik A., Byrne L., Scheinbaum M.L. (1991). Sibutramine in Weight Control: A Dose-Ranging, Efficacy Study. Clin. Pharmacol. Ther..

[77] Hanotin C., Thomas F., Jones S.P., Leutenegger E., Drouin P. (1997). Efficacy and Tolerability of Sibutramine in Obese Patients: A Dose-Ranging Study. Int. J. Obes..

[78] Bray G.A., Blackburn G.L., Ferguson J.M., Greenway F.L., Jain A.K., Mendel C.M., Mendels J., Ryan D.H., Schwartz S.L., Scheinbaum M.L. (1999). Sibutramine Produces Dose-Related Weight Loss. Obes. Res..

[79] Di Francesco V., Sacco T., Zamboni M., Bissoli L., Zoico E., Mazzali G., Minniti A., Salanitri T., Cancelli F., Bosello O. (2007). Weight Loss and Quality of Life Improvement in Obese Subjects Treated with Sibutramine: A Double-Blind Randomized Multicenter Study. Ann. Nutr. Metab..

[80] James W.P.T., Caterson I.D., Coutinho W., Finer N., van Gaal L.F., Maggioni A.P., Torp-Pedersen C., Sharma A.M., Shepherd G.M., Rode R.A. (2010). Effect of Sibutramine on Cardiovascular Outcomes in Overweight and Obese Subjects. N. Engl. J. Med..

[81] Berkowitz R.I., Fujioka K., Daniels S.R., Hoppin A.G., Owen S., Perry A.C., Sothern M.S., Renz C.L., Pirner M.A., Walch J.K. (2006). Effects of Sibutramine Treatment in Obese Adolescents: A Randomized Trial. Ann. Intern. Med..

[82] McNulty S.J., Ur E., Williams G. (2003). A Randomized Trial of Sibutramine in the Management of Obese Type 2 Diabetic Patients Treated With Metformin. Diabetes Care.

[83] Hauner H., Meier M., Wendland G., Kurscheid T., Lauterbach K. (2004). Weight Reduction by Sibutramine in Obese Subjects in Primary Care Medicine: The S.A.T. Study. Exp. Clin. Endocrinol. Diabetes.

[84] Dujovne C.A., Zavoral J.H., Rowe E., Mendel C.M. (2001). Effects of Sibutramine on Body Weight and Serum Lipids: A Double-Blind, Randomized, Placebo-Controlled Study in 322 Overweight and Obese Patients with Dyslipidemia. Am. Heart J..

[85] Martin C.K., Redman L.M., Zhang J., Sanchez M., Anderson C.M., Smith S.R., Ravussin E. (2011). Lorcaserin, a 5-HT(2C) Receptor Agonist, Reduces Body Weight by Decreasing Energy Intake without Influencing Energy Expenditure. J. Clin. Endocrinol. Metab..

[86] Smith S.R., Prosser W.A., Donahue D.J., Morgan M.E., Anderson C.M., Shanahan W.R. (2009). Lorcaserin (APD356), a Selective 5-HT_2C_ Agonist, Reduces Body Weight in Obese Men and Women. Obesity.

[87] Shaw Tronieri J., Wadden T.A., Berkowitz R.I., Chao A.M., Pearl R.L., Alamuddin N., Leonard S.M., Carvajal R., Bakizada Z.M., Pinkasavage E. (2018). A Randomized Trial of Lorcaserin and Lifestyle Counseling for Maintaining Weight Loss Achieved with a Low-Calorie Diet. Obesity.

[88] Bohula E.A., Wiviott S.D., McGuire D.K., Inzucchi S.E., Kuder J., Im K., Fanola C.L., Qamar A., Brown C., Budaj A. (2018). Cardiovascular Safety of Lorcaserin in Overweight or Obese Patients. N. Engl. J. Med..

[89] O’Neil P.M., Smith S.R., Weissman N.J., Fidler M.C., Sanchez M., Zhang J., Raether B., Anderson C.M., Shanahan W.R. (2012). Randomized Placebo-Controlled Clinical Trial of Lorcaserin for Weight Loss in Type 2 Diabetes Mellitus: The BLOOM-DM Study. Obesity.

[90] Smith S.R., Weissman N.J., Anderson C.M., Sanchez M., Chuang E., Stubbe S., Bays H., Shanahan W.R. (2010). Multicenter, Placebo-Controlled Trial of Lorcaserin for Weight Management. N. Engl. J. Med..

[91] Aronne L., Shanahan W., Fain R., Glicklich A., Soliman W., Li Y., Smith S. (2014). Safety and Efficacy of Lorcaserin: A Combined Analysis of the BLOOM and BLOSSOM Trials. Postgrad. Med..

[92] Connolly H.M., Crary J.L., McGoon M.D., Hensrud D.D., Edwards B.S., Edwards W.D., Schaff H.V. (1997). Valvular Heart Disease Associated with Fenfluramine-Phentermine. N. Engl. J. Med..

[93] Khan M.A., Herzog C.A., Peter J.V.S., Hartley G.G., Madlon-Kay R., Dick C.D., Asinger R.W., Vessey J.T. (1998). The Prevalence of Cardiac Valvular Insufficiency Assessed by Transthoracic Echocardiography in Obese Patients Treated with Appetite-Suppressant Drugs. N. Engl. J. Med..

[94] Kancherla M.K., Salti H.I., Mulderink T.A., Parker M., Bonow R.O., Mehlman D.J. (1999). Echocardiographic Prevalence of Mitral and/or Aortic Regurgitation in Patients Exposed to Either Fenfluramine-Phentermine Combination or to Dexfenfluramine. Am. J. Cardiol..

[95] Surapaneni P., Vinales K.L., Najib M.Q., Chaliki H.P. (2011). Valvular Heart Disease with the Use of Fenfluramine-Phentermine. Tex. Heart Inst. J..

[96] McMurray C., Bloomfield P., Miller H.C. (1986). Irreversible Pulmonary Hypertension after Treatment with Fenfluramine. Br. Med. J..

[97] Pouwels H., Smeets J., Cheriex E., Wouters E. (1990). Pulmonary Hypertension and Fenfluramine. Eur. Respir. J..

[98] Bang W.D., Kim J.Y., Yu H.T., Cho S.S., Jang J.Y., Oh C.M., Joung B., Chang H.J. (2010). Pulmonary Hypertension Associated with Use of Phentermine. Yonsei Med. J..

[99] Schoonjans A.S., Marchau F., Paelinck B.P., Lagae L., Gammaitoni A., Pringsheim M., Keane M.G., Ceulemans B. (2017). Cardiovascular Safety of Low-Dose Fenfluramine in Dravet Syndrome: A Review of Its Benefit-Risk Profile in a New Patient Population. Curr. Med. Res. Opin..

[100] Davis R., Faulds D. (1996). Dexfenfluramine. An Updated Review of Its Therapeutic Use in the Management of Obesity. Drugs.

[101] Fitzgerald L.W., Burn T.C., Brown B.S., Patterson J.P., Corjay M.H., Valentine P.A., Sun J.H., Link J.R., Abbaszade I., Hollis J.M. (2000). Possible Role of Valvular Serotonin 5-HT(2B) Receptors in the Cardiopathy Associated with Fenfluramine. Mol. Pharmacol..

[102] Seimon R.V., Espinoza D., Ivers L., Gebski V., Finer N., Legler U.F., Sharma A.M., James W.P.T., Coutinho W., Caterson I.D. (2014). Changes in Body Weight and Blood Pressure: Paradoxical Outcome Events in Overweight and Obese Subjects with Cardiovascular Disease. Int. J. Obes..

[103] Daniels S.R., Long B., Crow S., Styne D., Sothern M., Vargas-Rodriguez I., Harris L., Walch J., Jasinsky O., Cwik K. (2007). Cardiovascular Effects of Sibutramine in the Treatment of Obese Adolescents: Results of a Randomized, Double-Blind, Placebo-Controlled Study. Pediatrics.

[104] Torp-Pedersen C., Caterson I., Coutinho W., Finer N., van Gaal L., Maggioni A., Sharma A., Brisco W., Deaton R., Shepherd G. (2007). Cardiovascular Responses to Weight Management and Sibutramine in High-Risk Subjects: An Analysis from the SCOUT Trial. Eur. Heart J..

[105] Zannad F., Gille B., Grentzinger A., Bruntz J.F., Hammadi M., Boivin J.M., Hanotin C., Igau B., Drouin P. (2002). Effects of Sibutramine on Ventricular Dimensions and Heart Valves in Obese Patients during Weight Reduction. Am. Heart J..

[106] Gürsoy A., Erdoǧan M.F., Cin M.Ö., Cesur M., Başkal N. (2005). Effect of Sibutramine on Blood Pressure in Patients with Obesity and Well-Controlled Hypertension or Normotension. Endocr. Pract..

[107] De Simone G., Romano C., de Caprio C., Contaldo F., Salanitri T., di Luzio Paparatti U., Pasanisi F. (2005). Effects of Sibutramine-Induced Weight Loss on Cardiovascular System in Obese Subjects. Nutr. Metab. Cardiovasc. Dis..

[108] Heusser K., Engeli S., Tank J., Diedrich A., Wiesner S., Janke J., Luft F.C., Jordan J. (2007). Sympathetic Vasomotor Tone Determines Blood Pressure Response to Long-Term Sibutramine Treatment. J. Clin. Endocrinol. Metab..

[109] Merenich J.A. (2004). The Long-Term Outcomes of Sibutramine Effectiveness on Weight (LOSE Weight) Study: Evaluating the Role of Drug Therapy Within a Weight Management Program in a Group-Model Health Maintenance Organizati. Am. J. Manag. Care.

[110] Fanghänel G., Cortinas L., Sánchez-Reyes L., Berber A. (2000). A Clinical Trial of the Use of Sibutramine for the Treatment of Patients Suffering Essential Obesity. Int. J. Obes. Relat. Metab. Disord..

[111] Guven A., Koksal N., Cetinkaya A., Sokmen G., Ozdemir R. (2004). Effects of the Sibutramine Therapy on Pulmonary Artery Pressure in Obese Patients. Diabetes Obes. Metab..

[112] Saraç S., Saraç F. (2010). Cardiac Valve Evaluation and Adipokine Levels in Obese Women Treated with Sibutramine. Anadolu Kardiyol. Derg..

[113] Caterson I.D., Finer N., Coutinho W., van Gaal L.F., Maggioni A.P., Torp-Pedersen C., Sharma A.M., Legler U.F., Shepherd G.M., Rode R.A. (2012). Maintained Intentional Weight Loss Reduces Cardiovascular Outcomes: Results from the Sibutramine Cardiovascular OUTcomes (SCOUT) Trial. Diabetes Obes. Metab..

[114] Maggioni A.P., Caterson I., Coutinho W., Finer N., van Gaal L., Sharma A.M., Torp-Pedersen C., Bacher P., Shepherd G., Sun R. (2008). Tolerability of Sibutramine during a 6-Week Treatment Period in High-Risk Patients with Cardiovascular Disease and/or Diabetes: A Preliminary Analysis of the Sibutramine Cardiovascular Outcomes (SCOUT) Trial. J. Cardiovasc. Pharmacol..

[115] Weeke P., Andersson C., Fosbøl E.L., Brendorp B., Køber L., Sharma A.M., Finer N., James P.T., Caterson I.D., Rode R.A. (2010). The Weight Lowering Effect of Sibutramine and Its Impact on Serum Lipids in Cardiovascular High Risk Patients with and without Type 2 Diabetes Mellitus—An Analysis from the SCOUT Lead-in Period. BMC Endocr. Disord..

[116] Nisoli E., Carruba M.O. (2000). An Assessment of the Safety and Efficacy of Sibutramine, an Anti-Obesity Drug with a Novel Mechanism of Action. Obes. Rev..

[117] Tuccinardi D., Farr O.M., Upadhyay J., Oussaada S.M., Mathew H., Paschou S.A., Perakakis N., Koniaris A., Kelesidis T., Mantzoros C.S. (2019). Lorcaserin Treatment Decreases Body Weight and Reduces Cardiometabolic Risk Factors in Obese Adults: A Six-Month, Randomized, Placebo-Controlled, Double-Blind Clinical Trial. Diabetes Obes. Metab..

[118] Pi-Sunyer X., Shanahan W., Fain R., Ma T., Garvey W.T. (2016). Impact of Lorcaserin on Glycemic Control in Overweight and Obese Patients with Type 2 Diabetes: Analysis of Week 52 Responders and Nonresponders. Postgrad. Med..

[119] Bohula E.A., Scirica B.M., Inzucchi S.E., McGuire D.K., Keech A.C., Smith S.R., Kanevsky E., Murphy S.A., Leiter L.A., Dwyer J.P. (2018). Effect of Lorcaserin on Prevention and Remission of Type 2 Diabetes in Overweight and Obese Patients (CAMELLIA-TIMI 61): A Randomised, Placebo-Controlled Trial. Lancet.

[120] Fidler M.C., Sanchez M., Raether B., Weissman N.J., Smith S.R., Shanahan W.R., Anderson C.M. (2011). A One-Year Randomized Trial of Lorcaserin for Weight Loss in Obese and Overweight Adults: The BLOSSOM Trial. J. Clin. Endocrinol. Metab..

[121] Weissman N.J., Sanchez M., Koch G.G., Smith S.R., Shanahan W.R., Anderson C.M. (2013). Echocardiographic Assessment of Cardiac Valvular Regurgitation with Lorcaserin from Analysis of 3 Phase 3 Clinical Trials. Circ. Cardiovasc. Imaging.

[122] Greenway F.L., Shanahan W., Fain R., Ma T., Rubino D. (2016). Safety and Tolerability Review of Lorcaserin in Clinical Trials. Clin. Obes..

[123] Gorelik E., Gorelik B., Masarwa R., Perlman A., Hirsh-Raccah B., Matok I. (2020). The Cardiovascular Safety of Antiobesity Drugs-Analysis of Signals in the FDA Adverse Event Report System Database. Int. J. Obes..

[124] Scirica B.M., Bohula E.A., Dwyer J.P., Qamar A., Inzucchi S.E., McGuire D.K., Keech A.C., Smith S.R., Murphy S.A., Im K. (2019). Lorcaserin and Renal Outcomes in Obese and Overweight Patients in the CAMELLIA-TIMI 61 Trial. Circulation.

[125] Bray G.A. (2014). Medical Treatment of Obesity: The Past, the Present and the Future. Best Pract. Res. Clin. Gastroenterol..

[126] De Andrade Mesquita L., Fagundes Piccoli G., Richter da Natividade G., Frison Spiazzi B., Colpani V., Gerchman F. (2021). Is Lorcaserin Really Associated with Increased Risk of Cancer? A Systematic Review and Meta-Analysis. Obes. Rev..

[127] Sharretts J., Galescu O., Gomatam S., Andraca-Carrera E., Hampp C., Yanoff L. (2020). Cancer Risk Associated with Lorcaserin—The FDA’s Review of the CAMELLIA-TIMI 61 Trial. N. Engl. J. Med..

[128] Jeffrey Conn P., Christopoulos A., Lindsley C.W. (2009). Allosteric Modulators of GPCRs: A Novel Approach for the Treatment of CNS Disorders. Nat. Rev. Drug Discov..

[129] García-Cárceles J., Decara J.M., Vázquez-Villa H., Rodríguez R., Codesido E., Cruces J., Brea J., Loza M.I., Alén F., Botta J. (2017). A Positive Allosteric Modulator of the Serotonin 5-HT 2C Receptor for Obesity. J. Med. Chem..

[130] Singh K., Sona C., Ojha V., Singh M., Mishra A., Kumar A., Siddiqi M.I., Tripathi R.P., Yadav P.N. (2019). Identification of Dual Role of Piperazine-Linked Phenyl Cyclopropyl Methanone as Positive Allosteric Modulator of 5-HT_2C_ and Negative Allosteric Modulator of 5-HT_2B_ Receptors. Eur. J. Med. Chem..

[131] Cifuentes L., Eckel-Passow J., Acosta A. (2021). Precision Medicine for Obesity. Dig. Dis. Interv..

[132] Severin R., Sabbahi A., Mahmoud A.M., Arena R., Phillips S.A. (2019). Precision Medicine in Weight Loss and Healthy Living. Prog. Cardiovasc. Dis..

[133] Hurtado A.M.D., Acosta A. (2021). Precision Medicine and Obesity. Gastroenterol. Clin. N. Am..

[134] Griebsch N.I., Kern J., Hansen J., Rullmann M., Luthardt J., Helfmeyer S., Dekorsy F.J., Soeder M., Hankir M.K., Zientek F. (2022). Central Serotonin/Noradrenaline Transporter Availability and Treatment Success in Patients with Obesity. Brain Sci..

[135] He Y., Brouwers B., Liu H., Liu H., Lawler K., Mendes de Oliveira E., Lee D.K., Yang Y., Cox A.R., Keogh J.M. (2022). Human Loss-of-Function Variants in the Serotonin 2C Receptor Associated with Obesity and Maladaptive Behavior. Nat. Med..

[136] Wold E.A., Garcia E.J., Wild C.T., Miszkiel J.M., Soto C.A., Chen J., Pazdrak K., Fox R.G., Anastasio N.C., Cunningham K.A. (2020). Discovery of 4-Phenylpiperidine-2-Carboxamide Analogues as Serotonin 5-HT_2C_ Receptor-Positive Allosteric Modulators with Enhanced Drug-like Properties. J. Med. Chem..

